# An airlock concept to reduce contamination risks during the human exploration of Mars

**DOI:** 10.1038/s41526-023-00329-5

**Published:** 2023-10-07

**Authors:** Daniel Vrankar, Cyprien Verseux, Christiane Heinicke

**Affiliations:** 1 Faculty of Business and Economics, Technische Universität Dresden, Helmholtzstraße 10, 01069 Dresden, Germany; 2 Center of Applied Space Technology and Microgravity - ZARM, University of Bremen, Am Fallturm 2, 28359 Bremen, Germany

**Keywords:** Aerospace engineering, Environmental sciences, Ethics, Microbiology

## Abstract

Protecting the Martian environment from contamination with terrestrial microbes is generally seen as essential to the scientific exploration of Mars, especially when it comes to the search for indigenous life. However, while companies and space agencies aim at getting to Mars within ambitious timelines, the state-of-the-art planetary protection measures are only applicable to uncrewed spacecraft. With this paper, we attempt to reconcile these two conflicting goals: the human exploration of Mars and its protection from biological contamination. In our view, the one nominal mission activity that is most prone to introducing terrestrial microbes into the Martian environment is when humans leave their habitat to explore the Martian surface, if one were to use state-of-the-art airlocks. We therefore propose to adapt airlocks specifically to the goals of planetary protection. We suggest a concrete concept for such an adapted airlock, believing that only practical and implementable solutions will be followed by human explorers in the long run.

## Introduction

If there is or has been life on Mars, the presence of a crew on-site would bring unprecedented capabilities for its detection and study. At the same time, there is widespread concern that microbial contamination caused by humans on site could jeopardize the search for life—or even harm an indigenous ecosystem.

A milder version of this dilemma applies to robotic missions to Mars. For those, the Committee on Space Research (COSPAR) has defined pragmatic rules to minimize contamination risks without preventing exploration (Table 1)^1^. It has been argued, however, that those rules could not be applied to human missions^2,3^: crews will inevitably carry microbial populations and contaminate any environment directly exposed to them. How this could be handled remains to be fully determined: recommendations have been outlined^1,4^ but no operational processes have yet been detailed, or hardware developed, which would enable crewed missions to Mars that meet stringent cleanliness requirements.

**Table 1:** Overview of surface bioburden requirements for category IV missions (applicable to robotic missions with landing on Mars), as given by the 2021 COSPAR Policy on Planetary Protection^1^.

Category	Description	System level	Maximum bioburden (spores)	Maximum bioburden density (spores m^−2^)
IVa	No search for extant Martian life	Full system	3 × 10^5^	300
IVb	Search for extant Martian life	Full system, or subsystems involved in life detection	30, or as required by the nature and sensitivity of the life-detection experiments	3 × 10^−2^
IVc (1)	Investigations in Special Region, with landing site in Special Region	Full system	30	3 × 10^−2^
IVc (2)	Investigations in Special Region, with landing site outside Special Region	Full system, or subsystems in direct contact with the Special Region	30	3 × 10^−2^

The requirements apply to exposed or accessible spacecraft surfaces. “Spores” refer to aerobic microorganisms which survive a heat shock of 80 °C for 15 min and are cultured on TSA at 32 °C for 72 h. Special regions are areas within which terrestrial organisms are likely to replicate, or which have a high potential for hosting extant Martian life.

The principles and guidelines for human missions to Mars given by COSPAR’s Policy on Planetary protection^1^ include a statement that “planetary protection goals should not be relaxed to accommodate a human mission to Mars”. Whether those goals justify retaining the limits on bioburden defined for hardware, or whether a reassessment of the risks may lead to milder requirements, is still being debated. Deciding for the former would, it seems, mean giving up on crewed missions to Mars for the foreseeable future. At the other end of the debate, it has been argued that current planetary protection measures are excessive even for robotic missions^5^. An example of the arguments put forward is that either Mars can support terrestrial life, in which case the later has most likely colonized it already (after reaching it through natural panspermia or with contaminated spacecraft), or the biocidal factors in the Martian environment^6–9^ are lethal to any terrestrial life form and would promptly inactivate microbial contaminants.

While deciding on stringent bioburden requirements may, at first glance, seem like the safest option, it critically depends on compliance from the entities involved in space exploration. All major spacefaring countries are parties to the 1967 Outer Space Treaty, which is formally binding and whose Article IX stipulates that the harmful contamination of celestial bodies should be avoided; but, as pointed out by Fairén and Schulze-Makuch^5,10^, what constitutes “harmful contamination” (or what standards should be held) is not defined, which limits the guidance and constraints this rule provides. COSPAR is offering more detailed guidelines. It is, however, an advisory committee, and to date no country has integrated its recommendations into national law. Most entities involved in space exploration have been largely compliant so far but one cannot ascertain that this will remain the case. If planetary protection recommendations are seen as too restrictive, they may be passed over.

One more viable approach to this problem could lie in providing space actors with pragmatic solutions to accommodate planetary protection concerns. Strategic choices—e.g., avoiding the Special Regions (where terrestrial organisms are likely to replicate, or which have a high potential for hosting indigenous life), if some are identified, until adequately sterilized rovers have determined a near-absence of risk can be instrumental in meeting planetary protection goals, but they cannot in themselves ensure compliance to stringent bioburden requirements. Engineering solutions will be required. There is some urgency: dedicated technologies should be designed this decade if they are to stand a chance of being integrated into missions foreseen for the next.

Planetary protection technologies should, in particular, mitigate the risks posed by extravehicular activities (EVAs). Forward contamination could occur during egress, when aerosols from inside the habitat will be most prone to leaking, and during operations in a potentially contaminated suit. Conversely, backward contamination (the contamination of astronauts, or later of terrestrial ecosystems, by extant Martian life) would be most likely during ingress. Risks are exacerbated by the fact that scientific and maintenance needs of crewed missions to Mars are expected to lead to a much higher frequency of EVAs than in the current ISS program, increased from an average of around 10 EVAs a year to perhaps 3 to 6 a week^11^.

Various airlock designs have been relied on in the past decades^12^. None is suitable for a Martian habitat. First, because none were designed to avoid microbial contamination: that was not a requirement for the systems they have been part of. When an astronaut comes out of the ISS Quest Joint Airlock (today’s state-of-the-art airlock), for instance, about a pound of non-sterile air is vented out when opening the hatch, and astronauts carry large numbers of microorganisms on the outside of their suits^13^. Second, because no measures have been taken to minimize backward biological contamination. Third, because no effective measures against contamination with dust have been implemented (while backward contamination with dust was a concern in the Apollo surface missions, the issue was not solved to satisfaction). In fact, “[d]eveloping technologies for minimizing/mitigating contamination release, including [..] cleaning/re-cleaning capabilities” has been declared a Strategic Knowledge Gap by NASA for human missions to Mars^14^.

Here, we outline the design of an airlock for crewed missions to Mars, modified from today’s state-of-the-art to address planetary protection concerns. While the airlock could not, in itself, bring contamination risks as low as currently allowed for robotic missions—EVAs will not be the only possible cause for contamination—it would represent a large step toward reconciling planetary protection requirements and human exploration goals.

## Methods

**Fig. 1:**
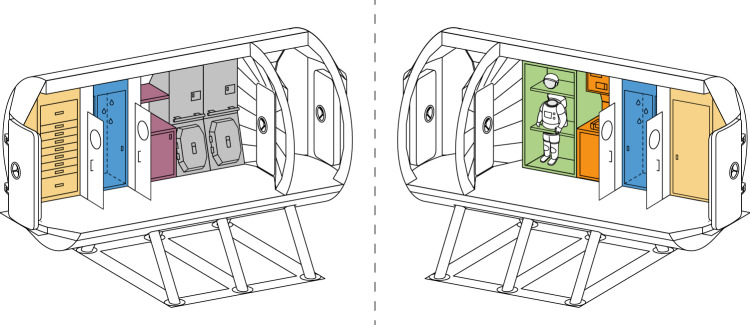
Artistic rendering of an airlock module (3.4 m diameter) that would fulfill the requirements described in “Methods”. The airlock proper is between the two pressure-tight doors. The remaining room is divided into three compartments or anterooms that are separated by airtight doors. From the center of the figure (Mars surface) towards the edges (the other habitat modules), the three compartments are: (1) suit-up area, suit repair, and stowage of EVA suits; possible location of suitports, (2) showers and general hygiene compartment, (3) stowage for undergarments and indoor clothes. Note that most of the functions of the airlock module would need to be present in the habitat anyway, but that the *SafeMars* concept combines those functions into one module that are helpful for planetary protection. Image by J. Wegner.

In this section, we outline our airlock concept *SafeMars,* and the five measures that we propose to considerably reduce forward contamination. Most of these measures are also effective against backward contamination. The airlock concept is depicted in Fig. 1.

### Five measures to counter contamination

The first measure is to divide the airlock into compartments of increasing “cleanliness” toward the living quarters. Before each transfer from one compartment to the next, the crew undertakes steps to reduce the level of bioburden on themselves and their gear (through showering, using protective clothing, etc.).

The second measure is to actively reduce the bioburden in the outer compartment, the airlock proper, through disinfection. Rather than using alcohol wipes and similarly resource-intensive methods, we propose to use a gaseous reagent on the outside of the surface suits.

Third, unlike on the ISS, where a portion of the station air is vented into space before each egress, the air in an airlock on Mars should be evacuated down to the pressure levels of the surrounding atmosphere, approximately 6 mbar. Preferably, the air is stored in separate tanks; if it is pumped into the remainder of the habitat, effective and reliable filters should be in place.

Fourth, effective dust mitigation measures should be implemented. Even though dust mitigation by itself is not mandated by planetary protection goals, most measures that reduce the amount of dust brought into the habitat will also help reduce the amount of biological matter on the outside of the surface suits.

Finally, the plain airlock could be supplemented with suitports, creating a combination that is commonly referred to as suitlock. In such a system (e.g., ref. ^11^), a suit is attached to the outside of the crewed compartment. An astronaut enters from the back, and then separates the suit from the crewed compartment to perform an EVA. Suitports could help minimize (not eliminate) the exchange of material between the inside and outside, as well as allow for rapid ingress and egress. In the following we describe *SafeMars* as a stand-alone airlock, although Fig. 1 shows where suitports could be integrated if need be.

In any case, while suitports are already under research and development, there is a gap for airlocks that could conform with planetary protection requirements. Hence, our work focuses on airlocks—be it as flexible, but resource-intensive, stand-alone systems, or as part of a suitlock.

## Results and discussion

In this section, we describe the first four measures in detail. For the fifth measure, suitports, we refer the reader to the numerous existing concepts (see, e.g., refs. ^15–18^).

### Compartmentalization

**Fig. 2:**
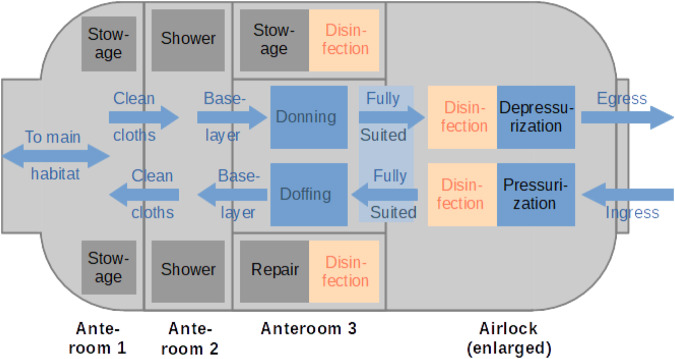
Schematic floorplan showing the general workflow during EVA preparation and post-EVA clean-up and servicing. The disinfection cycle inside the airlock is complemented by disinfection cycles inside separate compartments in Anteroom 3 for selected suit parts (e.g., interior of boots, poorly exposed parts of gloves, cuffs, etc.). The airlock is enlarged here for visualization purposes.

We suggest dividing the airlock module into four sections, similar to Biosafety level 3/4 (BSL-3/4) laboratories on Earth (see Fig. 1). Access to BSL-3/4 laboratories^19^ leads through (at least) three anterooms (see Fig. 2). In the first anteroom, personnel changes from street clothes to scrubs. Next, they enter the suit room where BSL-3/4 suits are inspected and donned. After a chemical shower in the third anteroom, the laboratory can be entered. Exiting procedures are similar but require an additional personal shower in Anteroom 1 before changing back into street clothes^20^. It should be noted that BSL-3/4 laboratories are effective in protecting personnel from pathogens, i.e., from the equivalent of *backward* contamination.

As our focus is on forward contamination, we modify the proposed layout for the *SafeMars* airlock module as such: The first anteroom remains for changing from the crew’s casual wear into scrubs. The second room contains a water shower (see below) where the crew changes into their EVA undergarments and disposable gloves. The EVA suits are stored in the third anteroom, where the crew suits up and afterward discards their gloves. After entering the final section, the airlock proper, the EVA suits are disinfected with a disinfecting agent, such as ozone (see “Disinfection agents” section below).

There is a slight pressure gradient between all anterooms, with the highest pressure in the airlock (when pressurized) in order to inhibit the spread of microbes from inside the habitat.

Upon returning from their EVA, the crew undergoes another disinfection cycle in the airlock. Suit components with small spaces which are difficult to reach, such as gloves and boots, can be disinfected in small compartments (flooded with ozone or another gaseous disinfectant) in Anteroom 3. After doffing their suits, the astronauts take a shower in Anteroom 2 before changing back into their casual wear. This configuration assumes that astronauts on Mars, contrary to their colleagues on the ISS, will have access to a shower regardless of airlock design. The *SafeMars* concept simply proposes that this shower (or the hygiene system selected in lieu of a shower) be located in the airlock module.

All module compartments should be separated by doors equipped with an interlocking mechanism^21^, such that any compartment can only be entered or exited on one side at any given time. For the size of the individual compartments, it should be noted that astronauts likely need help from other crew members donning and doffing their suits^22,23^, although help may not be possible in the case of a 2-person surface crew—as is envisioned in the latest update to NASA’s Moon to Mars Architecture^24^. One option for providing the relatively large volume inside the airlock module without driving up system mass could be the use of inflatables, if the compatibility of airlock subsystems with the inflatable hull can be ensured.

### Disinfection agents

Beside compartmentalization, active disinfection methods will be the most important tool to reduce the microbial burden down to reasonable limits. The exact limits are still up for debate and will likely depend on the target areas for both the habitat and EVAs, as well as the risk level of contamination deemed acceptable (typically, 10^−3^ during the period of exploration). Here we chose COSPAR’s current bioburden requirements for Category IVa missions (see Table 1) as a tentative reference, as we expect requirements for crewed missions to Mars to be less stringent than they currently are for uncrewed, search-for-life missions.

The current method of bioburden reduction for uncrewed spacecraft to Mars is physical cleaning with alcohol wipes^4,25^, which is time-consuming and resource-intensive. Given the high number of planned EVAs on Mars^11^, the *SafeMars* disinfection process should use a disinfectant that enables short process times and can be distributed highly efficiently, reaching creases and other surfaces of complex geometries. Furthermore, the ideal disinfectant is either reusable or can be produced directly on Mars, reducing the need for supply flights.

Table 2 provides an overview of disinfectants potentially suitable for the in-process decontamination of space suits. Given the problems associated with the other disinfectants and particularly their transport to Mars, we deem ozone (O_3_) the most promising option, possibly complemented with UV radiation.

**Table 2:** Overview of disinfectants commonly used in hospitals and potentially usable for the in-process decontamination of space suits.

Disinfectant/method	Advantages	Disadvantages
Physical cleaning using alcohol wipes^4,25^	Effective on surfaces; wipes could be re-used if autoclaved	Time-consuming; alcohol must (most likely) be brought from Earth; less effective for complex geometries
UV light^75,76^	Could be generated on-site; short process times (< 20 min)^77,78^; treatment of various kinds of surfaces and ambient air	Less effective when no direct line of sight
Ozone	Could be generated on-site; decays fairly quickly, and half-life can be reduced further; increases sensitivity for UV light; chemically removes microbes; treatment of various kinds of surfaces and ambient air	Less effective on porous materials (e.g. textiles); toxic; most effective with high temperature and relative humidity; strong oxidant; chemical interaction with certain materials
Vapor phase H_2_O_2_	Approved by ESA and NASA as microbial reduction method^4^	Supply (most likely) needs to be brought from Earth; known bacterial resistances in spacecraft assembly clean rooms^79^; strong oxidant; chemical interaction with certain materials
Other gas-phase chemicals^80,81^	Commonly used in the medical industry; treatment of ambient air and surfaces	Toxic; chemical interaction with certain materials
Liquid chemicals (aerosols)^82^	Very effective on surfaces	Supply needs to be brought from Earth; not all materials can be treated directly; leave residues
Supercritical or liquid CO_2_^83,84^	High efficacy; could be produced from in situ resources	Scalability unclear; does not reach into crevices; limited material compatibility

Ozone can be produced on-site and chemically removes microbes from surfaces. This genotoxic gas decays with a short half-life (approx. 30–40 min at 25 °C^26,27^) but has a reduced efficacy on porous materials like textiles^28^. It affects bacteria^29,30^, spores^29,31^, viruses^32,33^—including bacteriophages^34,35^—and fungi^36^. The damage it causes is a combination of damage to the cell wall^37^, damage to the capsid (protein shell of a virus)^38^, damage to amino acids^36,38^, and damage to DNA/RNA^37^, although what effect is most relevant is not yet known^32,37^. Ozone also enhances the sensitivity of microbes to UV light^37^.

The efficacy of ozone depends on its exposure, which is the product of ozone concentration and exposure time^38,39^ (unit ppmmin or ppmm), with the reduction of the microbial load decreasing exponentially with time^38,40,41^. An ozone concentration of ~8000 ppmm at ambient air pressure is expected to be sufficient for a log-2 reduction in the bioburden^29,42^. To reach the limit on microbial load recommended by the COSPAR IVa category, an initial bioburden below 3 × 10^7^ spores total and 3 × 10^4^ spores/m^2^ would be acceptable (for reference: the microbial load in ISO 8 clean rooms as used for planetary protection purposes is < 50 spores/m^2^ (see ref. ^43^)). Assuming a (conservative) ozone concentration of 300 ppm, the duration of the disinfection process would be ~27 min if this ozone concentration could be reached instantaneously, ozone decayed instantaneously (or had no adverse health effects) and there were no changes in total pressure (see “Gas management” section below).

In reality, the ozone disinfection process by itself consists of three phases that take a total of 97 min. It should be emphasized that this duration refers to the disinfection alone and ignores any pressure changes, which will be dealt with in the next section. In the *SafeMars* concept, the disinfection cycle overlaps with the de-/repressurization cycle such that astronauts do not actually spend 97 min for disinfection plus ca. 40 min for de-/repressurization inside the airlock.

**Fig. 3:**
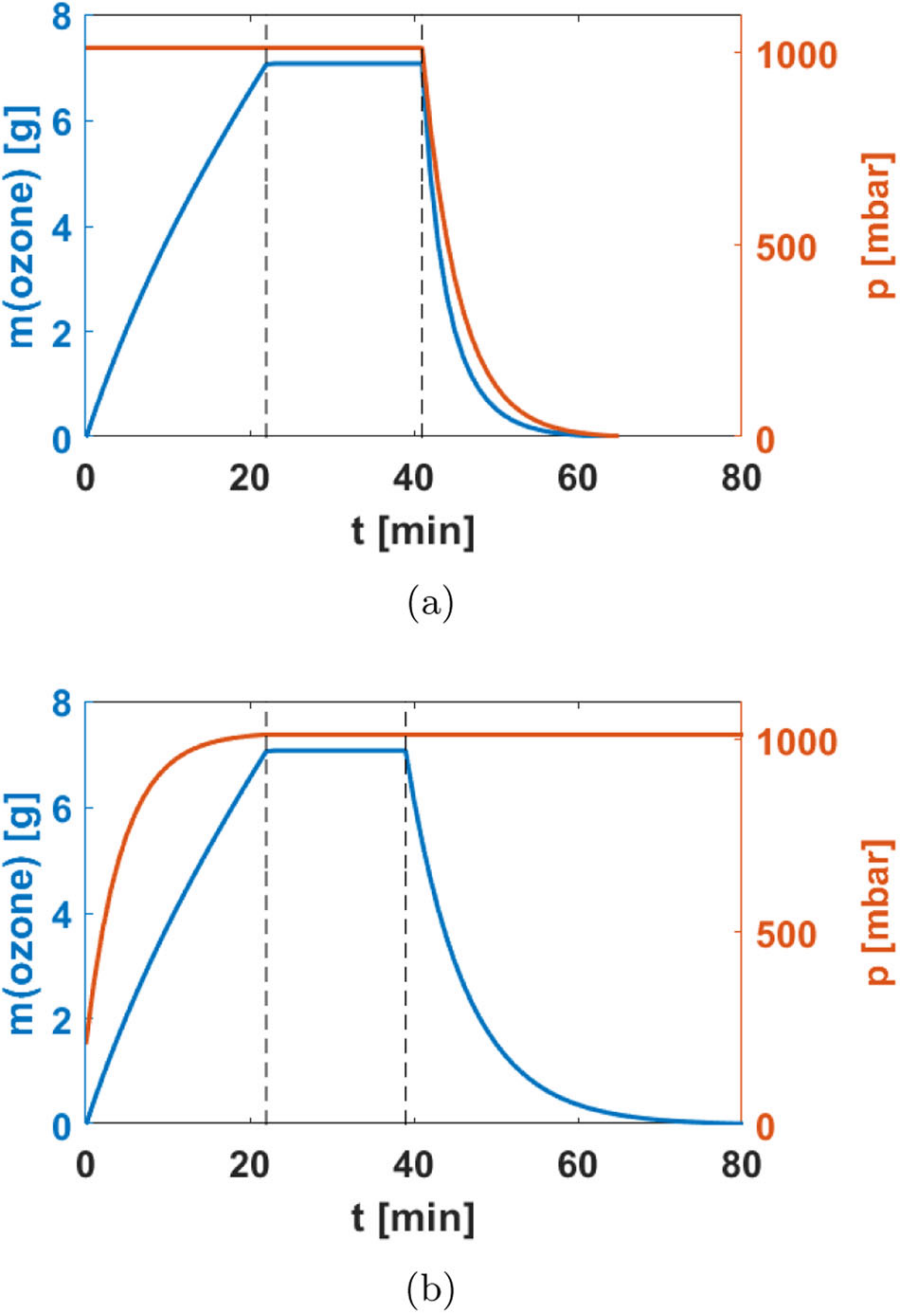
Model of the locking process showing ozone total mass (blue) and total pressure (red). **a** Evacuation and egress. The phase of constant ozone concentration has been extended to account for the fast decline in phase 3 due to the evacuation of the airlock air. **b** Ingress and repressurization. The rate of ozone production depends on the production rate of the ozone generator; the decay of ozone can be accelerated with separate pumps or catalysts. In the depicted conservative scenario, the total time is 64 min for egress and 97 min for ingress. The target exposure of ozone is 8000 ppmm at 300 ppm, the final pressure before egress is Mars atmospheric pressure of 6 mbar, and the reference pump is a SOGEVAC 200.

The three phases of disinfection are (see Fig. 3):

#### Ozone generation

Typical ozone generators produce between 5 and ~30 g/h of ozone, although high-power generators that use pure oxygen can reach up to 50 g/h. As an example, for a generation of 28 g/h, ozone should be produced for 22 min until the target concentration of 300 ppm has been reached.

#### Constant ozone concentration

The target concentration should be kept constant for 17 min, to reach a total exposure (over all three disinfection phases) of 8000 ppmm.

#### Ozone decay

The decay is exponential, but determining the half-life of ozone is not trivial and the few empirical models only work for very specific applications^42^. In experiments, the half-life of ozone has ranged from slightly more than 5 min^26,44,45^ to 20–50 min^26,46^. The influence of the half-time on reaching the target concentration is significantly smaller than the production rate of the generator^42^. The ozone concentration is largely relevant for ingress; if one assumes a half-life of 5 min, it takes 58 min for the ozone concentration to drop to levels that are usually deemed safe for humans^37^.

In total, the time per EVA for disinfection is 64 min (egress) and 97 min (ingress). For comparison, the current depressurization procedures on the ISS take around 50 min^47^, not including prebreathe protocols which add another 460 min to an EVA^11^. It should also be noted that the duration indicated for our concept does not pertain to disinfection only: decompression and repressurization also occur within that time. Finally, our estimates are highly conservative: First, because actual constraints on bioburden will most likely be much below the reference used here and second, because our calculations are based on commercially available ozone generators rather than devices which could be developed specifically.

The efficacy of ozone can be enhanced: high relative humidity increases its efficacy through the formation of aggressive chemicals^27,32,37,39,48^, and so does high temperature^36,49–51^. However, both also accelerate the decay of ozone. The net effect remains positive, i.e., higher temperatures and humidity may lead to a lower concentration of ozone but still cause a faster inactivation of microorganisms^49,51^.

Values around 30 °C and 70%, which are the maximum values for the Temperature and Humidity Control sub-assembly on the ISS, seem achievable; perhaps a more up-to-date and powerful life support systems could handle higher values. A gas ballast in the vacuum pump could allow for even higher humidity when evacuating the airlock.

Another possible option to reduce the overall disinfection time would be to combine different disinfectants, like gaseous chemicals and UV light (see, for instance, though for sterilization in a liquid, Koivunen and Heinonen-Tanski^52^).

Finally, we would like to stress that, while ozone currently appears most promising, other gaseous disinfectants (see Table 2) should not be disregarded altogether at this stage: The selection of materials in the airlock depends on the disinfectant, and certain natural rubbers or metals are susceptible to oxidation induced by ozone unless treated^38,53^ or coated with PTFE, which is resistant to it. As the literature on ozone susceptibility of specific materials is sparse, prolonged exposure tests for ozone (or any other gaseous disinfectant) would be needed.

### Gas management

As explained above, previous airlocks are not suitable for the surface of Mars. One of the most recent systems, the ISS Quest Joint Airlock module installed in 2001, can recover 90% of the air during the locking process. During depressurization, most of the air is pumped into the crew compartment of the Unity module. Once 34 mbar have been reached^47^, the remaining air is vented into space. The depressurization time is between 30 and 40 min^54^.

Compared to low Earth orbit, re-supply missions to Mars are drastically more costly and take longer. More importantly, simply venting the airlock air into the Martian atmosphere would contaminate the latter with terrestrial organic substances. Hence, on Mars, the pressure inside the airlock must at least be reduced to Mars ambient pressure, ~6 mbar^55^. At the same time, pumping duration should remain within bearable limits, and preferably below the duration of the Quest module as it is expected to be used much more frequently.

In any case, the most obvious way of reducing the amount of habitat gas leaking into the Martian atmosphere through the airlock is reducing airlock volume. A volume sufficient for at least two suited astronauts is considered the bare minimum^56,57^. For reference, the volume of the Crew Lock of the Quest module is 8.8 m^3^.

The volume of the *SafeMars* airlock is 8.5 m^3^, and this amount of air must be stored during EVA. If the air is not pumped back into the habitat, as is done for the Quest module, separate containers must be provided. One option is to use three separate containers of the same size as the airlock, which are filled one after the other during depressurization^54^. This concept would allow for fast and energy-conservative transfer of air, but would require the shipment of 3 either relatively large tanks or inflatable structures to Mars. The former option only seems feasible if such tanks can be constructed in situ.

**Fig. 4:**
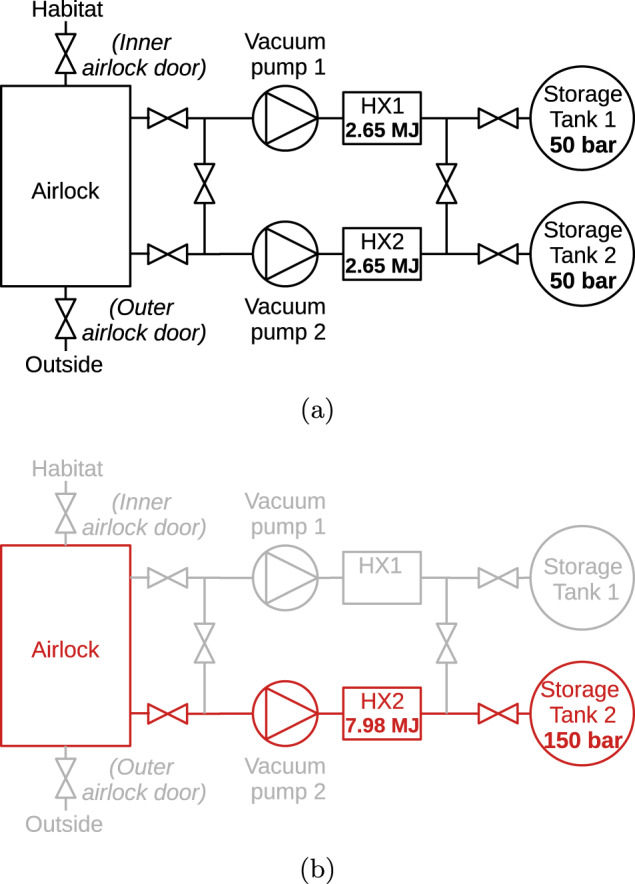
Gas system layout. **a** Nominal operation with two storage tanks that hold air at 50 bar and two heat exchangers for a total 5.3 MJ of generated heat. **b** If one storage system fails, the other needs to hold 140 bar and handle up to 7.98 MJ^58^.

A more practical alternative is to store the air in pressure tanks, ideally two for redundancy (see Fig. 4). At 140 bar, each tank has a volume of 0.2 m^3^. In normal operation (see Fig. 4, center), both are filled with filtered^4,14^ airlock air at 50 bar. This generates a heat of 5.3 MJ^58^, necessitating a heat exchanger for each tank. Using both tanks not only reduces the operating pressure but also generates less heat, the remainder of which can be dissipated by the heat exchangers, and greatly reduces the fatigue loads on the components, thereby increasing their service life. The heat exchangers could possibly be coupled with the cold Martian atmosphere, which would further reduce its energy consumption. However, given the low atmospheric density, convective heat transfer alone is unlikely to make the heat exchanger dispensable.

In emergency operation (see Fig. 4, bottom), and only then, the full capacity of the system is reached by filling only one tank with the entire airlock air and using a single heat exchanger to dissipate the heat generated. In this operation, the pressure in the single tank will be 140 bar and the generated heat 7.98 MJ^58^ (at the standard pressure of 300 bar, the temperature of the adiabatically compressed gas would reach over 1496 K, which most likely would lead to material failure^59^). The tanks and the heat exchange system must be designed for extended emergency operation.

The power consumption of the *SafeMars* airlock is estimated at 4.4 kWh for an airlocking process of 20 min^58^. By comparison, the Quest module needs only ~500–660 Wh.

A pressure of 6 mbar could be achieved with a single type of pump. One could consider evacuating the airlock to even lower pressures with another pump—and let the airlock fill with Martian atmosphere upon egress. For ingress, the Martian atmosphere could then be pumped outside, before the airlock is re-pressurized with habitat air. This process would reduce (though not remove entirely) the amount of residual air inside the airlock, but at the expense of being extremely time-consuming.

During evacuation, the pressure decreases exponentially with time, and so does the partial pressure of ozone. In order to reach the target exposure of 8000 ppmm, the phase of constant ozone concentration (300 ppm) must be extended by ~2 min to a total of 19 min. At the end of evacuation, the remaining ozone amounts to only 0.02 g—which can be vented into the Martian atmosphere for decay. Depending on the type of vacuum pump, the total locking process (disinfection plus evacuation) would last ~64 min. This estimate is rather conservative: in practice, most of the disinfection could be done at a pressure slightly lower than 1 bar, requiring either higher concentrations of ozone or longer exposure times, but allowing the crew to conduct any leak tests.

For ingress, the ozone decay period cannot be skipped and the entire disinfection process would consequently last 97 min. However, the decay of ozone could be accelerated with, e.g., catalysts^29,38^, so the total time required for disinfection before ingress could be similar as for egress.

### Dust mitigation

Martian dust has long been expected to pose challenges to technical systems (e.g., moving parts; solar panels) during exploration missions^60–62^. Inside the habitat, it may be harmful to human health due to perchlorates^63^ or chromium^64^ it contains. Mitigating contamination with dust is hence a goal in its own right. In addition, however, mitigating dust contamination helps mitigate the risk of microbial contamination, as most mechanisms effective with the former also contribute to the latter. One should therefore aim to, first, avoid direct contact with dust wherever feasible, and second, implement measures to remove dust from surfaces where it has settled anyways, such as suits, boots, and any parts of the airlock.

One effective measure for avoiding contact with dust lies in suitably prepared infrastructure, such as through sintering^65–67^ pathways and streets into the Martian regolith, reducing the contact with loose particles during activities in the vicinity of the habitat. However, this measure is only applicable to long-term stays on Mars and would not be suitable for early or exploration-type missions.

After contact, dust may be prevented from remaining on a surface by Lotus coating of part of the suits and airlock (see, e.g., Straka et al.^68^), electrostatic dust removal^69^ or an electrodynamic dust shield^70^. For suits, carbon nanotube fibers integrated into the outer layer seem promising^71^ as a passive measure.

Removing dust from the suit with manual methods, such as the use of brushes, did not work well during Apollo missions. An alternative could be the use of vacuum cleaners with HEPA and/or ULPA filters^72^ or pressurized fluids; gaseous CO_2_, in particular, would be abundantly available. Electron beams^73^ or magnetic cleaners^74^, as proposed for use with lunar regolith, are either extremely time-consuming or unlikely to be applicable to Martian regolith.

Overall, dust mitigation remains an open challenge. Upcoming Moon missions are expected to provide opportunities for testing at least some of the above measures. For *SafeMars*, a hybrid approach is suggested, consisting of passive measures (coating on hard surfaces, carbon nanotube or otherwise reinforced fabrics for soft surfaces) and active measures (electrostatic or electrodynamic dust removal for large surfaces and vacuum cleaners for remaining spots). This approach must of course be re-evaluated after the next Moon landings, especially regarding the cost/benefit ratio of each measure.

### Integration into habitat

We propose the *SafeMars* airlock concept as a tool for humans to access the Martian surface from their habitat, while simultaneously lowering the risk of biological contamination. The main focus of *SafeMars* is the forward contamination of Mars, but it also reduces the risk of backward contamination, albeit to a lesser extent (for example, the overpressure in Anteroom 3 is effective to prevent outgoing contamination but could increase the risk of incoming contamination if microbes made it that far).

The *SafeMars* concept allows for the integration of suitports (as is shown in Fig. 1). While suitports could by-pass the airlock proper for most EVAs, their peripheral systems require a relatively large volume, and they would not suffice as stand-alone systems for habitats (they might still be the preferred option for exploration vehicles). The longer the stay on Mars, the more a full-sized airlock is needed: maintenance and repairs on the suit can hardly be performed outside the habitat, and suitports limit the number and combination of crew members who can participate in an EVA at the same time, which may be problematic if more than two crew members are present on the surface. A suitlock not only allows for more flexibility, the airlock is also the safer option in the case of an incapacitated crew member or other type of emergency.

A number of questions still need to be answered for the final design of the airlock (or suitlock) module. Among them are the final selection of the disinfectant, or combination of disinfectants, and possible ways to increase their efficacy or reduce the required exposure times; the optimal characteristics of the inlet flow of the disinfectants, especially in combination with the inlets and outlets of the gas management system; the selection of materials for the airlock and its subsystems; and the optimization of the disinfection and de-/repressurization phases. Finally, testing the overall procedure of EVA preparation is crucial to ensure that the process is acceptable from a human factors perspective.

In any case, (nominal) EVAs are not the only possible source of contamination. The *SafeMars* airlock may help reduce the risk of forward contamination due to humans leaving their habitat. However, the habitat, and even more so the EVA suits, are likely to leak substantial amounts of microbes even during nominal operations. Hence, it is important to embed *SafeMars* into a more comprehensive concept for addressing the conflict between planetary protection goals and the human exploration of Mars.

## Data Availability

The disinfection model is described in detail in ref. ^42^, which is available online.
